# Defining synthetic surfaces for human pluripotent stem cell culture

**DOI:** 10.1186/2045-9769-2-7

**Published:** 2013-11-22

**Authors:** Jack W Lambshead, Laurence Meagher, Carmel O’Brien, Andrew L Laslett

**Affiliations:** 1 CSIRO Materials Science and Engineering, Clayton, Victoria 3168 Australia; 2 Australian Regenerative Medicine Institute, Monash University, Kragujevac, Victoria 3800 Australia; 3 Department of Zoology, University of Melbourne, Parkville, Victoria 3101 Australia

**Keywords:** Human embryonic stem cells, Induced pluripotent stem cells, Cell adhesion molecules, Pluripotency

## Abstract

Human pluripotent stem cells (hPSCs) are able to self-renew indefinitely and to differentiate into all adult cell types. hPSCs therefore show potential for application to drug screening, disease modelling and cellular therapies. In order to meet this potential, culture conditions must be developed that are consistent, defined, scalable, free of animal products and that facilitate stable self-renewal of hPSCs. Several culture surfaces have recently been reported to meet many of these criteria although none of them have been widely implemented by the stem cell community due to issues with validation, reliability and expense. Most hPSC culture surfaces have been derived from extracellular matrix proteins (ECMPs) and their cell adhesion molecule (CAM) binding motifs. Elucidating the CAM-mediated cell-surface interactions that are essential for the *in vitro* maintenance of pluripotency will facilitate the optimisation of hPSC culture surfaces. Reports indicate that hPSC cultures can be supported by cell-surface interactions through certain CAM subtypes but not by others. This review summarises the recent reports of defined surfaces for hPSC culture and focuses on the CAMs and ECMPs involved.

## Introduction

**Figure 1:**
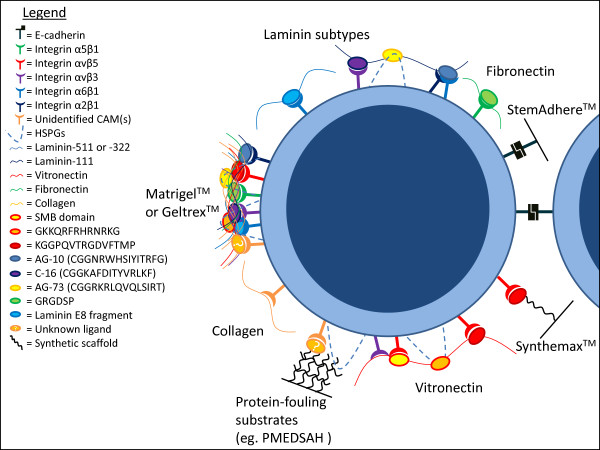
**Molecular interactions between human pluripotent stem cells (hPSCs) and culture surfaces.** A schematic diagram of a single hPSC illustrates molecular interactions with reported hPSC culture surfaces through different ligands and CAM subtypes. Specific ligands and cell adhesion molecules (CAMs) are included if they have been reported in hPSC attachment and/or culture studies. CAMs involved in hPSC adhesion include integrin subtypes α5β1 (green), αvβ5 (red), αvβ3 (purple), α6β1 (blue) and α2β1 (navy blue), E-cadherin (black blocks), heparan sulphate proteoglycans (HSPGs; dashed blue lines) and unidentified CAMs (orange). Ligands are portrayed as coloured ovals and include the SMB domain of vitronectin (yellow/red), GKKQRFRHRNRKG (orange/red), KGGPQVTRGDVFTMP (red/dark red), AG-10 (CGGNRWHSIYITRFG; blue/dark blue), C-16 (CGGKAFDITYVRLKF; purple/navy blue), AG-73 (CGGRKRLQVQLSIRT; yellow/orange), GRGDSP (green) and laminin E8 fragments (light blue/blue). The ligands are presented by ECMPs [represented by curved coloured lines: laminin-511 or −322 (blue), laminin-111 (navy blue), vitronectin (red), fibronectin (green) collagen (yellow)] or synthetic surfaces (thick black lines) including Synthemax^TM^, StemAdhere^TM^ and PMEDSAH. On the left of the image complex extracellular matrix extracts (eg. Matrigel^TM^ and Geltrex^TM^) are illustrated as combinations of ECMPs, and on the right cell-cell adhesion is simplified in the extreme to illustrate homophilic E-cadherin binding. Where specific ECMP ligands are poorly-defined, CAMs are shown to interact with the ECMP line. Where specific CAMs have not been identified the orange CAM is used, and undefined, adsorbed ligands are represented by orange ovals with a white question mark. This figure is a greatly simplified and stylised representation of the cell-surface and cell-cell adhesion interactions important for hPSCs and discussed in this review.

Human pluripotent stem cells (hPSCs) include embryonic stem cells (ESCs) and induced pluripotent stem cells (iPSCs) and have enormous potential for applications to drug screening, disease modelling and cellular therapies [1, 2]. These applications will necessitate the use of cell culture conditions that are consistent, chemically-defined and/or non-xenogenic for reasons of scale, reproducibility and safety. hPSCs are adherent cells and have long been cultured on poorly-defined, complex surfaces of xenogenic origin. Such surfaces present a wide range of ligands and interact with hPSCs via poorly understood mechanisms through many different cell adhesion molecules (CAMs) on the cell surface. CAM-ligand interactions are restricted by the types of CAMs and ligands available and are governed by the physical properties of the culture surface. Specific CAM-ligand interactions mediate various intracellular signalling pathways thought to be involved in maintaining the homeostasis and self-renewal of hPSCs. The involvement of CAM-mediated intracellular signalling pathways in the maintenance of hPSCs are addressed in the following reviews [3–5]. A detailed understanding of the effects of CAM-surface interactions on hPSC phenotype and behaviour in culture should facilitate the optimisation of defined culture conditions to support both hPSC self-renewal and somatic differentiation pathways. A wide variety of chemically-defined surfaces that engage different CAM subtypes have been reported to support the long-term self renewal of hPSCs [for examples [6–13]. It is challenging to elucidate the roles of CAMs from these reports due to the diverse physicochemical properties of the culture surfaces as well as the inter-laboratory variation in cell culture protocols and in the cell and surface characterisation methods utilised. Non-specific protein adsorption to many “defined” surfaces can also confound results [14]. Direct comparisons between culture surfaces and the hPSCs cultured thereon are limited and have been focussed on identifying systems able to support culture of hPSCs as defined by minimal criteria including gene expression and qualitative differentiation assays [15–17]. Detailed characterisation and direct comparison of hPSCs cultured on defined surfaces that specifically engage different CAMs is required to elucidate the roles of CAMs in maintaining pluripotency. The following review describes published reports of defined culture surfaces for hPSC self-renewal with a focus on the CAMs and extracellular matrix proteins (ECMPs) thought to be involved in mediating cell-surface interactions and maintaining pluripotency (Figure 1).

### Human pluripotent stem cells

Pluripotency describes the ability of single cells to differentiate into every cell type in the developing and adult body [18]. Pluripotent stem cells are also capable of indefinite self-renewal *in vitro* under appropriate conditions. hPSCs are therefore a potential cell source for myriad regenerative medicine approaches and *in vitro* disease models, for example hPSC-derived cardiomyocytes could be used to repair damaged tissue following a myocardial infarction [1, 2]. Pluripotency is a complex state that is maintained *in vitro* by large transcriptional networks that are yet to be fully elucidated [reviewed by [19]. Although many genes are involved in the regulation of pluripotency, cell line variation and population heterogeneity have hampered the identification of reliable molecular markers of pluripotency [20, 21]. To further complicate matters, murine studies have identified multiple pluripotent states that are maintained by different signalling networks [22]. It has been suggested that many of the differences between murine pluripotent stem cells (mPSCs) and hPSCs could be attributed to mPSC and hPSC cultures representing different states of pluripotency and that hPSCs can move between these states with changes in culture conditions [22, 23]. All of these factors make correct identification and characterisation of hPSCs a challenging task. Adequate characterisation of hPSCs is essential for the unambiguous identification of surfaces capable of supporting hPSC expansion.

### hPSC characterisation methods

**Figure 2:**
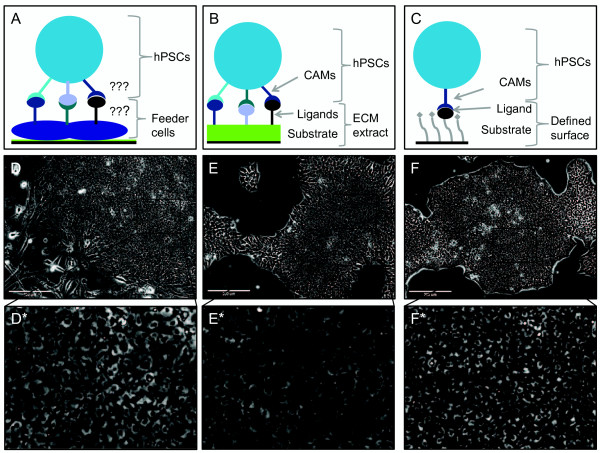
**HPSCs cultured on different surfaces.** Schematic diagrams illustrate the arrangement of cell adhesion molecules (CAMs), ligands and substrates (where appropriate) of the three major types of culture surfaces used for maintenance of human pluripotent stem cells (hPSCs). **(A)** Feeder cells, **(B)** extracellular matrix (ECM) extracts and **(C)** chemically defined culture surfaces. **(D-F)** Phase contrast images of hPSCs cultured on one example of each surface type, murine embryonic fibroblasts, Geltrex^TM^ and Corning Synthemax^TM^ respectively. (D*-F*) Magnified regions of D-F. hPSCs cultured on each surface display a typical morphology with compact colonies of cells with prominent nucleoli and high nuclear-to-cytoplasmic ratio.

The quality of ongoing hPSC cultures should be regularly assessed. When developing or implementing novel culture conditions it is important to characterise the cells thoroughly in order to validate the culture system. Daily assessment of hPSC cultures should involve visual observation of characteristic tightly-packed colonies of cuboidal-shaped cells containing prominent nuclei, multiple nucleoli and little cytoplasm, with minimal differentiated cell types present as shown in Figure 2[1]. Proliferation rates of ongoing cultures can be monitored over time by recording approximate cell seeding densities and the frequency of passaging, but when comparing various culture conditions the proliferation rate should be calculated more accurately from serial cell counts of ongoing cultures at multiple time points. Stronger evidence for pluripotency can be generated by monitoring associated molecular markers. The gold standard genetic marker of pluripotency is POU domain, class 5, transcription factor 1 (Pou5f1) aka OCT4, a homeodomain transcription factor of the POU family that is essential for pluripotent cells [24]. Expression of OCT4 and other markers can be assessed in populations of hPSCs using numerous methods, listed in Table 1[25–28]. Additional information about the cell state can be obtained by characterising the epigenetic signature. Epigenetic regulation of gene expression is exercised through modifications to the genome that do not affect the genetic sequence. DNA methylation is one of the most-studied epigenetic modifications. Methylation down-regulates expression of local genes and can be detected by sequencing bisulfite-treated DNA [29]. Signature methylation patterns can be used to identify developmentally regulated cell types and individual hPSC lines and change in response to environmental stimuli [reviewed by [30]. DNA methylation patterns have also been linked to the differentiation potential of hPSCs and can therefore be used as molecular markers of pluripotency [31]. Molecular markers are however not completely specific to pluripotent cells due to the inherent heterogeneity of hPSCs. For example subpopulations with reduced differentiation potential have been identified within OCT4-positive populations of hPSCs [21]. While combinatorial assessment of marker expression improves the robustness of molecular assays for pluripotency they ultimately remain surrogate assays, whereas functional demonstrations of cell potential provide more stringent tests of pluripotency. The ability of hPSCs to differentiate into cell types of all three embryonic germ layers (endoderm, ectoderm and mesoderm) can be examined both *in vitro* and *in vivo. In vitro* differentiation of pluripotent cells is usually associated with the formation of embryoid bodies [complex, non-adherent, three-dimensional structures composed of spontaneously differentiating hPSCs [32, 33] and can either be spontaneous or directed towards certain cell fates [25, 34]. The *in vivo* differentiation potential of hPSCs is typically tested by transplantation into immunodeficient mice. The formation of a teratoma (a benign tumour comprising cell types representative of each of the three embryonic germ layers) at the site of implantation is the most stringent validation assay available for the differentiation potential of putative hPSCs [18]. However, differentiation assays are laborious, inconsistent in efficiency and difficult to standardise across cell lines and laboratories, so evaluation of molecular markers remains important for assessing the efficacy of hPSC culture systems. Quality control of any long-term cell culture system should also include an assessment of genetic stability using G-banding analysis to detect gross or subchromosomal changes. However, genetic aberrations below the detection limit of G-banding have been identified in hPSC lines and more detailed genetic analysis should also be considered when testing novel culture systems [35]. A detailed characterisation of hPSCs should include the methodologies bolded in Table 1.

**Table 1:** **Parameters of interest for hPSC characterisation and methods for their assessment**

Parameters	Method	Strength of evidence of pluripotency
Physical characteristics (daily/weekly)	**Daily visual assessment of cell/colony morphology**	Weak, subjective
	calculate adhesion efficiency, population doubling time	
Expression of molecular markers eg. **OCT4**, NANOG, SOX2, REX1 (following passages 1, 5 and >10)	**Immunocytochemical staining**, flow cytometry, RT-PCR, microarray assays	Moderate-strong. Depending on marker(s) assessed.
Epigenetic profiling	Bisulfite sequencing, ChIP, microarray assays	Moderate-strong. Depending on marker(s) assessed.
Differentiation potential (following >10 passages)	Embryoid body differentiation (*in vitro*) with RT-PCR analysis for molecular markers of differentiation	Very strong
	**Teratoma formation assay** (*in vivo*) with histological determination of cells from the three embryonic germ layers	Gold standard
Genetic stability (following >10 passages)	**G-banding,** FISH, SNP analysis	Not applicable. Important to identify genetically transformed cultures, not indicative of differentiation potential

Physical characteristics, molecular markers, epigenetic profiling, differentiation potential and genetic stability can be assessed by the range of methods listed (not comprehensive). We recommend the methods highlighted in bold performed at frequencies indicated in the first column as the minimum requirements for validating novel culture systems. Unbolded methods should also be considered for more thorough characterisation of hPSCs.

### The evolution of hPSC culture surfaces

It is well known that *in vitro* maintenance of the pluripotent state requires culture in supportive media within a favourable cellular microenvironment. An important aspect of the cellular microenvironment is the culture surface, to which hPSCs are anchored by CAMs. hPSCs are routinely cultured in vessels containing complex media and coated with complex surfaces. The signalling pathways regulated by growth factors in the media and by ligands on the culture surface converge downstream and contribute to the maintenance of pluripotency, so the combination of surface and media is critical. When the cells reach confluence (usually after 4–7 days) they are enzymatically dissociated, lifted into suspension and a portion is transferred into freshly coated vessels to which they adhere and continue proliferating indefinitely. The most widely used systems for maintaining hPSCs persist from the early days of hPSC derivation and rely on a layer of either mitotically-inactivated mouse embryonic fibroblast (MEF) feeder cells (see Figure 2 A and D) or complex extracellular matrix (ECM) extracts including Matrigel^TM^ and Geltrex^TM^ (Figure 2 B and E) [1, 36]. These pluripotency-supporting materials are not compatible with large scale cultures, risk the introduction of pathogens, show batch-to-batch variability and interact with hPSCs in a poorly-defined way. On the other hand most practical applications of hPSCs, including potential use in cell therapies, will demand chemically-defined, xeno-free culture conditions. Many approaches to improving hPSC culture surfaces: using human or autologous feeders [37, 38], complex human ECM extracts [39], defined surfaces in combination with conditioned media (CM) [media imbued with factors secreted by cultured cells, often MEFs (MEF-CM)] [40] or even fixed MEFs [41] incompletely address these concerns. A suite of chemically-defined, xeno-free surfaces (see Figure 2 C and F) have recently been reported to support hPSC culture in defined media, although many of these surfaces are expensive and none have been widely employed by the stem cell community [9, 11, 42–44]. Defined surfaces that are thought to specifically interact with different CAM-subtypes have been reported to support hPSC culture (see Figure 1) but the roles of CAMs in maintaining pluripotency are poorly understood. hPSC culture surfaces must be identified that are reproducible, stable, xeno-free, affordable and that can be tailored to a range of long-term differentiation protocols. Such surfaces should be based on an understanding of the properties of the surfaces and of the requirements of the cells, including CAM-mediated signalling.

### Cell adhesion molecules (CAMs) and the maintenance of pluripotency

Cell adhesion molecules (CAMs) are cell-surface proteins that mediate interactions with nearby cells and ECMPs through extracellular ligands. When engaged by a ligand, CAMs transfer molecular signals “outside-in” to the nucleus of the cell resulting in modification of gene expression. Signals can also be transferred “inside-out” when cytoplasmic agonists alter the affinity of CAMs for certain ligands [reviewed by [45]. A range of CAMs have been considered as potential molecular markers for hPSCs and could be involved in the maintenance of pluripotency [46]. The main CAM families thought to be involved in hPSC maintenance are integrins and cadherins, which have each been shown to modulate self-renewal of hPSCs in culture on different surfaces [23].

#### Integrins

Integrins are a family of transmembrane heterodimeric glycoproteins that are composed of α and β chains. Eighteen α and eight β chains have been identified in humans, combining to form the twenty-four known types of integrin [reviewed by [3]. Different integrin types recognise and bind a range of ligands with various affinities. Bound integrins assemble at the cell surface and interact with numerous cytoplasmic proteins to form focal adhesions, which act as transmembrane signalling conduits regulating intracellular kinases and phosphatases [3]. The formation of focal adhesions by various integrin types modulates different downstream signalling pathways that mediate a range of cell responses and functions. In particular, roles in inner cell mass formation and cell survival make integrins promising targets for hPSC culture surfaces [47]. Integrins α5β1 [48], α6β1 [11], αvβ3 and αvβ5 [42] have been reported to mediate hPSC interactions with several defined culture surfaces and are thought to be involved in maintaining pluripotency.

#### Cadherins

Cadherins are transmembrane glycoproteins that form calcium-dependent cell-cell and cell-ECM homophilic binding junctions [reviewed by [4]. While the cadherin family comprises more than 100 members, E-cadherin is the primary cadherin expressed by hPSCs and its expression and engagement is important for hPSC function [49]. Active E-cadherin interacts with multiple intracellular signalling mechanisms and is involved in tissue morphogenesis, hPSC self-renewal [4] and hPSC mechanosensing of surface nanotopography [50]. Conversely, disruption of E-cadherin signalling has been linked to hPSC death following dissociation [23]. Accordingly, reduction in E-cadherin expression correlates with early differentiation processes *in vitro*[28]. E-cadherin clearly plays an important role in pluripotency and is therefore a target CAM for defined hPSC culture surfaces. As a homophilic binding protein, E-cadherin not only serves as a CAM for hPSC binding but also as a potential ligand. A recombinant human fusion protein composed of E-cadherin and the Fc region of IgG1 antibodies has been developed as a tissue culture coating for untreated polystyrene [10]. This fusion protein coating, which mediates cellular adhesion through the E-cadherin component, has been reported to support long-term culture of teratoma-forming hPSCs in combination with a range of chemically defined media and has been commercialised as StemAdhere^TM^[10, 43].

### Extracellular matrix proteins (ECMPs) as ligands for hPSC culture surfaces

Most defined culture surfaces have been developed though a reductive approach. Proteomic analyses of complex culture surfaces like MEFs and Matrigel^TM^ have identified several ECMPs involved in hPSC culture including collagen, laminin, fibronectin, vitronectin and heparan sulphate proteoglycans (HSPGs) [51, 52]. The ability of these ECMPs and their derivative peptides or molecular mimics, alone or in combination, to maintain hPSC culture is discussed below. Peptides and molecular mimics are of particular interest because they mediate fewer interactions than whole ECMPs, facilitating identification of key interactions.

#### Collagens

Collagens are large, trimeric proteins that assemble into fibrils and fibres and comprise the primary structural component of the extracellular matrix [reviewed by [53]. Collagens contain multiple binding sites that interact with a wide range of extracellular and cell surface proteins thought to be linked to pluripotency, including various integrins and HSPGs [reviewed by [54]. These binding sites and interactions suggest that collagen may be a suitable surface for hPSC culture; however in practice the demonstrated ability of collagen to support hPSCs has been limited. In a 5-day culture experiment collagen subtypes I, III and IV were able to support adhesion and proliferation of hPSCs in MEF-CM, although proliferation did not occur consistently on all subtypes or with all cell lines [15]. In longer-term studies MEF-CM (but not defined medium) has been shown to support OCT4 positive hPSC colonies on collagen IV for at least five passages [7], but not on collagen I due to poor attachment [40]. Medium that was conditioned by cells derived from an embryoid-body culture was able to support OCT4 positive hPSC colonies on collagen I for at least one month [40]. In the latter study, media conditioned by two other somatic cell types failed to support hPSC culture beyond two passages on Matrigel^TM^, highlighting the considerable effects that undefined components in CM can have on the effectiveness of a culture system and the importance of defined media for achieving reproducible cultures. Inclusion of collagen-derived peptides in cell culture surfaces has not been reported and the CAMs involved in the hPSC-collagen interactions have not been investigated, probably due to the lack of success in maintaining cultures on whole collagen. Curiously, long term culture of hPSCs has been achieved on gelatin (a derivative of collagen) in heavily-supplemented serum-free medium that was designed to emulate mPSC culture conditions [23]. These “converted hPSCs” could self-renew for more than 20 passages, were OCT4 positive by immunostaining and demonstrated the ability to differentiate into cell types of the three embryonic germ layers *in vitro*. The morphology and gene expression of converted hPSCs was reported to be more mPSC-like and the CAMs involved in mediating adhesion shifted from predominantly integrins to E-cadherin [23]. This unique example raises questions about the phenotypic stability and adaptability of hPSCs in various culture conditions and demonstrates the importance of standardisation of culture media.

#### Laminins

Laminins are cruciform, trimeric ECMPs composed of α, β and γ chains and are major proteins in the basal lamina. There are 5α, 4β and 3γ chains that combine to form the 15 known laminin subtypes. Subtypes are named for their chain composition, for example laminin-511 is composed of α5 β1 and γ1 chains. Laminin proteins have been proposed as hPSC culture surfaces due to their prominence in more complex surfaces [52] and because they contain multiple cell-binding motifs including RGD, E8, IKVAV, AG-10, C-16 and AG-73 [55, 56] that interact with various integrins and proteoglycans expressed by hPSCs [57]. Mixed-subtype human laminin has maintained self-renewing, teratoma-forming hPSCs in several conditioned and defined media over long-term culture and during the establishment of hESC cell lines [58, 59]. Five of the fifteen laminin subtypes have since been tested as hPSC culture surfaces coupled with the use of MEF-CM. In two independent studies laminins-511, -322 and −111 (which all interact with integrin α6β1) supported culture of OCT4 positive hPSCs capable of *in vitro* differentiation into the three germ layers for at least 10 passages, while laminins-211 (α3β1) and −411 (α7β1) failed to even support adhesion [11, 60]. Laminin-511 is the most thoroughly tested subtype and has been demonstrated to support long-term hPSC culture in three different chemically-defined media and further to support hESC derivation [11, 61].

Several peptides derived from laminin-111 have been incorporated into hPSC culture surfaces with varying results. In adhesion studies a peptide (RNIAEIIKDI) derived from the γ-chain successfully mediated attachment of hPSCs in defined xeno-free media while an IKVAV-containing peptide bound hPSCs poorly [9, 62]. Other laminin-111-derived peptides (AG-10, C-16, AG-73) have mediated adhesion of hPSCs in defined media by engaging different integrin subtypes or heparan sulphates [57]. These small peptides were only able to support hPSCs for a few passages when presented together in specific proportions, with differentiation apparent by the third passage [57].

More recently, larger laminin fragments composed of three post-translationally modified polypeptide subunits that form the E8 binding sites [63] of laminins-332 and −511 have been reported to support the long term culture of teratoma-forming hPSCs in a range of defined media [13]. E8 fragments demonstrated a stronger adhesion affinity to hPSCs than either Matrigel^TM^ or the whole laminins from which they were derived, which is a promising finding for the application of functional subunits to replace whole ECMPs in defined surfaces for hPSC culture [13]. The mixed reports of hPSC culture on laminin-derived subunits reflect how hPSCs can attach to different binding sites on single ECMPs with different affinities and that the maintenance of pluripotency depends on properties of CAM-mediated interactions with the culture surface beyond simple adherence.

#### Fibronectin

Fibronectin subunits are large (~250 kDa) extracellular glycoproteins which form disulfide-bonded dimers and much larger fibrils [64]. Fibronectin is ubiquitously expressed throughout the developing and adult body and plays an organisational role in assembling other ECMPs [reviewed by [65]. In theory fibronectin is a promising candidate surface for hPSC culture because it contains many binding domains that interact with ECMPs and CAMs associated with the self-renewal of hPSCs including fibrin, collagen, heparan sulfates and integrins [65]. In practice fibronectin has been favourably compared to laminin for its ability to support hPSC adherence and maintenance of pluripotency [15, 66]. Whole fibronectin has been reported to maintain long term cultures of OCT4 positive hPSCs capable of *in vitro* differentiation into the three germ layers and/or teratoma formation in MEF-conditioned [66] or heavily supplemented serum-free media [48] and also in several chemically-defined media [6, 67, 68].

Fibronectin-cell interactions are predominantly mediated by the GRGDSP motif and its interaction with α5β1 integrins [69]; the key role of α5β1 integrins in hPSC-fibronectin adhesion has been supported by a competitive inhibition study [48]. Nevertheless, defined surfaces that presented two fibronectin-derived, GRGDSP-containing, α5β1-binding peptides gave poor hPSC adhesion, suggesting that the ability of fibronectin to support hPSC culture relies on multiple binding domains [9]. A larger fusion peptide-amphiphile developed by Mardilovich et al., [70] and containing a GRGDSP motif has shown comparable adhesion and specificity to fibronectin in endothelial cell culture, although the ability of this peptide to support hPSCs in long term culture has not been reported [70]. Considering the success of whole fibronectin as a hPSC culture surface it remains of interest to determine whether this and other fibronectin-derived peptides can support the maintenance of hPSCs.

#### Vitronectin

Human vitronectin is a relatively small (75 kDa) glycoprotein that can be secreted as either a single chain or a dimer, is abundant in blood and throughout the ECM and promotes cell adhesion and migration [reviewed by [71]. Vitronectin contains multiple binding sites that engage integrins or HSPGs, which positions vitronectin as one of the most promising ECMPs for hPSC culture [72]. The potential applications of vitronectin to hPSC culture are exemplified by a vitronectin-containing chimeric protein that has been used as a media supplement for hPSC culture on a laminin-coated surface [61]. Vitronectin-coated tissue culture polystyrene (TCP) has supported long-term culture of teratoma-forming hPSCs [7, 12, 73, 74] and hiPSC derivation [75, 76] in a range of conditioned and defined media. Only two groups have reported the failure of vitronectin-based surfaces to maintain the pluripotency of cultured hPSCs in appropriate media and these results were probably due to inadequate deposition of the vitronectin protein [16, 77]. Hakala et al., [16] were unable to maintain hPSC culture on vitronectin-coated TCP and Abraham et al., [77] on a surface composed of vitronectin and HSPG (vitronectin-alone was not tested). Both studies used lower concentrations of vitronectin (200 ng/cm^2^ and 10 ng/cm^2^ respectively) than the threshold for hPSC culture of 250 ng/cm^2^ that was later determined by Yap et al., [73]. Since neither study reports surface characterisation such as performed by Yap et al., [73] we must assume that concentrations were calculated based on the vitronectin concentration in solution and assuming 100% deposition. Thus the actual protein surface concentrations in these studies would have been well below the threshold, explaining the inability of these culture systems to maintain hPSCs.

As implied by the binding sites described above, hPSC-vitronectin adhesion is mediated by αvβ3/β5 integrins and HSPGs [8, 42]. Accordingly, two integrin-binding peptides and one HSPG-binding peptide derived from vitronectin have been individually demonstrated to support long-term culture of teratoma-forming hPSCs [8, 9, 78]. The subtype-specificity of the integrin-binding peptides was not assessed, although anchored small molecules that specifically bind αvβ3 integrins have failed to support hPSC adhesion [79]. The latter small-molecule data is difficult to interpret without side-by-side and combinatorial comparisons with other integrin-specific small molecules. The apparent functional redundancy within the vitronectin molecule has demonstrated that small peptides can be sufficient to mimic the hPSC-supporting effects of ECMPs and suggests that the maintenance of pluripotency may not depend entirely on specific CAM-ligand interactions.

#### Combinations of ECMPs as hPSC culture surfaces

Combinations of collagens, fibronectin, laminins and vitronectin have been reported to support long-term culture of hPSCs in a few studies [15, 80], although combining ECMPs in this way can be superfluous given the aforementioned abilities of single-ECMPs (especially vitronectin and laminin-511) to support hPSCs. In one study, hPSCs cultured on a fibronectin-coated surface became differentiated after fewer than five passages while a fibronectin + HSPG surface supported culture of alkaline phosphatase-positive cells without morphological differentiation [77]. The initial experiments were conducted in a serum-free medium that had been supplemented with 4 ng/ml basic fibroblast growth factor (FGF-2) and although cells proliferated on fibronectin-HSPG in this medium and expressed alkaline phosphatase they would not differentiate when induced [77]. In the same study the culture on fibronectin + HSPG was repeated using media supplemented with additional FGF-2 (100 ng/ml) and the maintenance of pluripotency was demonstrated by successful embryoid body formation. It is unclear if the fibronectin-alone culture was repeated with the higher concentration of FGF-2 in this study [77]. These results contrast with other reports of hPSCs being maintained long-term on fibronectin using defined media and highlight the need for investigation of key signalling pathways involved in the maintenance of pluripotency and the importance of standardisation of defined culture methods [48, 66, 68],81].

#### Surfaces that interact with hPSCs through non-specific adsorbed media proteins

Some surfaces capable of supporting hPSC culture contain no obvious ligands; these surfaces usually rely on a level of culture medium and/or hPSC-secreted protein adsorption (protein-fouling) to mediate adhesion [14, 82]. Although protein-fouling surfaces may lack native ligands they often have modifiable physical properties which can be used to control cell behaviour and to optimise the adsorption of protein or peptide ligands (discussed further below). In one unusual report, Bigdeli et al., [83] reported the culture of two hPSC lines directly on TCP in a neonatal chondrocyte-conditioned medium (NC-CM). Media conditioned by several different somatic cell types were trialled, but only NC-CM supported effective adhesion and ongoing culture [83]. It would be interesting to determine which factor secreted by the neonatal chondrocytes was critical to this result. Adaptation to these culture conditions involved high levels of cell death and required an unusually long (20-day) recovery period. Nevertheless the cells were maintained long-term, thoroughly characterised for gene expression and differentiation potential and no genetic abnormalities were detected in either cell line by G-banding or FISH analysis. However, culturing hPSCs directly onto polystyrene has not been reproduced by other groups and the hPSC lines used (SA167 and AS034.1) have not been reported in any other culture surface studies and may behave uniquely [83].

While protein-fouling surfaces may adequately support hPSC culture they cannot be used to investigate critical molecular signalling pathways due to their poorly defined cell-surface interactions. Another consideration for these surfaces is that the type and concentration of adsorbing proteins may vary between cell lines and media formulations. A surface that depends on adsorbed proteins to mediate adhesion may be vulnerable to these variations and is likely to vary in its effectiveness at supporting different cell lines [84].

### Two-dimensional substrates for hPSC culture surfaces

In many of the aforementioned culture systems, the CAM-engaging proteins or peptides are simply applied to TCP in aqueous solutions from which they adsorb onto the plastic e.g. [7, 57]. However physicochemical modifications to TCP and more complex substrates including self-assembled monolayers (SAMs), polymer scaffolds and hydrogels are also being developed to optimise surface properties including hardness [85], roughness [50], stiffness, wettability [42] and ligand density [8], distribution [9] and presentation [86]. Some alterations to the physical properties of culture substrates {eg. nanoroughness [50]} are thought to be recognised directly by hPSCs while others such as wettability affect the adsorption and presentation of media and secreted proteins [87], indirectly influencing CAM-mediated cell-surface interactions.

### Characterisation methods for hPSC culture surfaces

In order to optimise hPSC culture surfaces the physical characteristics of the surfaces must be tuned to optimise CAM-ligand interactions. The effects of modulation of various physical properties on hPSC culture and detailed descriptions of methods to characterise these properties are beyond the scope of this review. In lieu of that, Table 2 is intended to create awareness in cellular biologists of the range of methods available and appropriate for this non-biological but very important aspect of developing synthetic culture surfaces for hPSC culture. It is worth noting here that it remains a challenge to control and characterise the quantity, distribution and orientation of ligands bound to polymer surfaces. Since these properties influence CAM-ligand interactions it is of interest to the field to develop new technologies to evaluate them. Throughout the following discussion on hPSC culture substrates, a “substrate” is defined as the non-ligand parts of a surface, those parts that position and present ligands rather than directly engaging CAMs.

**Table 2:** **Parameters of interest for characterisation of hPSC culture surfaces and analytical methods for their assessment**

Parameter	Analysis method	Pros	Cons
Surface topography	Atomic force microscopy (tapping mode)	Compatible with an aqueous environment, can view individual proteins that have absorbed to the surface, modern instruments acquire images at a faster rate.	Images are generally of a small area, therefore may not be representative.
	Scanning or transmission electron microscopy	Widely available	Resolution is not as high, significant sample preparation is required, unable to quantify topography.
Ligand density	ELISA assays	Straightforward assay	Not very sensitive for adsorbed protein, requires antibodies to specific proteins or molecules.
	Fluorescence from adsorbed or covalently attached fluorophore	Relatively straightforward assay	Microenvironment and dye-dye quenching effects from surface anchored species introduces artefacts, construction of calibration curve difficult.
	Fluorescence from fluorophore released into solution	Quantitative, sensitive, relatively straightforward assay	Cleavable fluorophore needs to be synthesised and chemically attached to ligand/CAM.
	Lanthanide (e.g. Eu-chelate) labelling of ligand	Quantitative, sensitive, relatively straightforward assay	Need to carry out chemical coupling of Eu-chelate to ligand.
	Radio-labelling of ligand	Quantitative, sensitive, relatively straightforward assay	Complex chemistry required to either radio-label pre-synthesised ligands or synthesise ligand with radioisotope-containing precursors.
Chemical properties	Nuclear magnetic resonance (NMR)	Straightforward sample preparation	Solid-state NMR generally not sensitive enough, complex spectra.
Wettability	Water contact angle	Simple	Very non-specific - many adsorbed species can modify wettability,
Chemical composition (directly detecting protein adsorption)	X-ray photoelectron spectrometry	Elemental composition quantitative, sample preparation is very simple (removal of buffer salts and drying).	Elemental composition is straightforward but high resolution spectra complex, amide bond-containing materials generate false positives, no specificity in relation to protein type, ultra high vacuum technique (can cause structural rearrangements).
	Time-of-flight secondary ion mass spectrometry	Minimally-destructive, minimal sample preparation, efficient,	Analysis generally not quantitative, produces large data sets often requiring statistical methods, no specificity in relation to protein type, ultra high vacuum technique (can cause structural rearrangements)
	Fourier transform infrared spectroscopy	Widely available, can be powerful if coupled with synchrotron	Not “surface-sensitive” enough, no specificity in relation to protein type.
Indirect assessment of protein adsorption	Embryoid body adhesion assay	Straightforward if embryoid bodies are being generated in house	Expensive, time-consuming
	HeLa or other e.g. L929 cell adhesion assay	Reliable, cheap if cell lines are available in laboratory	Cell attachment for cells other than hPSCs may be mediated by different ligands.

Surface topography, ligand density, chemical properties, wettability and protein adsorption can be tested by the methods listed. Pros and cons are listed for each method in this non-comprehensive list.

#### Modifications to plastic and glass culture surfaces

Simple modifications that have been used to make TCP or glass more biologically relevant for hPSC culture include amine-modifications [86] and physicochemical damage caused by UV-treatment [12], plasma-etching [82] or reactive ion etching [50]. These modifications increase the hydrophilicity, change the nanoscale topography and/or increase the abundance of functional groups on the surface, which can all modulate cell-surface interactions either through modulation of protein adsorption or by allowing modification with specific ligands.

#### Self assembled monolayers (SAMs)

Biologically-relevant self assembled monolayers (SAMs) are typically comprised of derivatives of organic alkanethiol (AT) molecules, which spontaneously form a monolayer on gold films comprehensively [reviewed by [88]. The thiol head groups bind to the gold film and the distal alkane tails arrange themselves roughly perpendicular to the film. Micropatterning of ATs with modified carboxyl or hydroxyl tail groups (to present ligands) and perfluoro-ATs (to provide a protein low-fouling background) allows presentation of multiple and varied ligands in particular orientations at controlled densities and distributions [88]. These customised AT-SAMs have been used by several groups to culture hPSCs and to investigate the molecular mechanisms involved in adhesion and maintenance of hPSCs. AT-SAMs have been used extensively by the Kiessling laboratory (University of Wisconsin) where laminin- and vitronectin-derived peptides have been identified that can support short and long term hPSC culture respectively [8, 62]. Micropatterned AT-SAMs have also been used to regionalise adsorption of mixed ECMPs onto carboxyl groups presented in a certain distribution, allowing fine control of hPSC colony size for medium-term (5 passages) culture [89]. Culturing hPSCs on these ECMP-islands resulted in colonies that were more homogenous for pluripotency marker expression than MEF-supported control cultures [89].

Although the customisable nature of SAMs makes them useful for investigating cell adhesion mechanisms, SAMs are physically unstable under biological conditions, which limits their utility for long-term hPSC culture and subsequent differentiation assays [90]. The gold coatings on which SAMs assemble can also present a challenge for visual assessment of live cultures [88]. For these reasons SAMs are unsuitable for practical applications as a long-term culture surface and while there is much to be learned from hPSC culture on SAMs it is currently unclear how transferrable results from SAM-based studies will be to other prospective culture surfaces.

#### Heparan sulfate proteoglycans (HSPGs)

Proteoglycans are large, membrane-bound extracellular proteins with covalently attached chains of repeating disaccharide units called glycosaminoglycans (GAGs). Proteoglycans are named according to GAG classes such that heparan sulphate proteoglycans (HSPGs) include all proteins bound to heparan sulphate polysaccharides {reviewed by Kim *et al*[3]}. Different proteoglycan subtypes are found throughout the mammalian ECM, but HSPGs are the most relevant to hPSCs and can be exploited in hPSC culture systems [8, 91, 92]. HSPGs bind to, stabilise and mediate interactions with integrins expressed by hPSCs [93] and with growth factors and their receptors. One such growth factor is FGF-2 [94], a key component of hPSC media which can mediate hPSC adhesion when immobilised [15]. This cooperative role of HSPGs is thought to explain why higher levels of FGF-2 are required to support feeder-free hPSC cultures when using non-conditioned media [95]. Since HSPG-hPSC interactions are often mediated by non-HSPG ligands (eg. FGF-2), for the purposes of investigating the molecular mechanisms involved in maintaining pluripotency HSPGs can be considered as a complex substrate that optimises presentation of a poorly-defined group of ligands.

Few studies have tested the effects of HSPGs on hPSC maintenance and differentiation. HSPGs have been shown to play a role in the differentiation of mPSCs and are not necessary for maintenance of their pluripotency [96]. However given mPSCs (unlike hPSCs) do not require FGF-2 signalling to self-renew [97] and predominantly interact with surfaces through different CAMs to hPSCs [23], this finding is not likely to be relevant to culture of conventional hPSCs. HSPG-mediated binding has not been able to support hPSC attachment on its own, although in combination with fibronectin HSPGs have improved the maintenance of pluripotency in the presence of higher concentrations of FGF-2, as discussed above [77]. The need for increased FGF-2 supplementation suggests that the HSPGs were not effectively presenting FGF-2 in this study or that other inadequacies in the culture system need to be compensated for [77].

The ability of heparan sulphate (HS) disaccharides to support hPSC culture has not yet been tested, although HSPG-binding peptides [8] and HS-mimicking polymers including poly(sodium 4-styrenesulfonate) (PSS(S)) and poly[2-(methacryloyloxy)ethyl dimethyl-(3-sulfopropyl)ammonium hydroxide] (PMEDSAH) have been used to successfully maintain long-term hPSC culture.

#### Polymer scaffolds

Polymer scaffolds can be loosely defined as physical networks composed of any polymer (long chains composed of monomer subunits). Polymer scaffolds can be applied to culture surfaces of various dimensions and their physicochemical properties can be tailored to meet the demands of hPSC cultures [42]. Polymers can interact with hPSCs due to intrinsic bioactivity or because they have been modified to present specific ligands. In the latter case, when investigating cell-surface interactions protein low-fouling polymers are preferred to minimize non-specific interactions with proteins and cells. Polymer scaffolds can also be employed as a base with controllable physical properties and then simply pre-coated with ECMPs [42].

The bioactive polymer on which hPSC culture has been most heavily studied is the zwitterionic PMEDSAH, which has been reported to support long-term hPSC culture in a range of conditions but performs most consistently in combination with various CM [44, 84, 98, 99]. The carboxyl and sulfonyl groups in PMEDSAH have been suggested to mimic HSPGs and to act as a reservoir for growth factors including FGF-2; however this hypothesis has not been tested by surface characterisation [100]. The reported protein low-fouling properties of zwitterionic materials that has led to their application to implantable medical devices only further confounds possible mechanisms for PMEDSAH-hPSC interaction [101]. Reports of PMEDSAH supporting hPSC attachment and culture suggest that PMEDSAH is not protein low-fouling and it will be treated as such for the remainder of this discussion. In serum-free chemically-defined media PMEDSAH can be an unreliable surface for hPSC culture and the ability of culture systems to maintain hPSCs depends on the media and cell-lines [98]. This inconsistency may be due to lower levels of certain proteins in defined media limiting the abundance of adsorbed ligands available for CAM binding. Culture results can be improved with pre-incubation of plates with FGF-2-supplemented (4 ng/ml) “human cell” CM (hCCM, GlobalStem®) and hCCM is also required during adaptation from MEF-based culture [44]. Other sulfonyl-containing polymers trialled as hPSC culture surfaces include PSS(S), poly(methyl vinyl ether-alt-maleic anhydride) (PMVE-alt-MA), poly(acrylamido-methyl-propane sulfonate) (PAMPS) and poly[3-sulfopropyl methacrylate] [98, 100]. PMVE-alt-MA has supported medium-term culture of several hPSC lines in a defined medium while PAMPS and PSS(S) enabled attachment but were unable to support hPSC culture beyond a few passages [100]. PSS(S) has also been integrated into a hPSC-supportive hydrogel which is discussed below [102]. Bioactive polymers that do not contain sulfonyl have been used to support hPSC culture when coated with ECMPs. Poly (lactic-co-glycolic acid) (PLGA) coated with subtype non-specific laminin has been reported to adhere hPSCs [103] while other bioactive polymers have been able to maintain teratoma-forming hPSCs in long-term culture when coated with either vitronectin or human serum [42, 104]. Although bioactive polymers may provide adequate support for hPSC culture they cannot be used to study cell-surface interactions because the interactions are mediated by adsorbed proteins, which can vary between media-type and cell lines.

Protein low-fouling polymer scaffolds with covalently attached ligands have not yet been reported to support hPSC culture. Protein low-fouling polymer brushes are promising substrates for investigating cell-surface interactions because they can be used to present specific ligands at a controlled density and in specific orientations. Polymer chains can be terminally-modified with different peptides using a range of simple chemistries including “click” chemistries and the formation of amide bonds. These chemistries can be tailored to specific peptides and performed under biologically-relevant conditions, allowing the peptides to retain functionality [reviewed by [105]. A low background of protein adsorption can be achieved by adjusting the density and length of the polymer chain “bristles” [106] while spacers of different molecular lengths ensure appropriate presentation of peptide ligands [107]. Importantly, polymer scaffolds are optically transparent which allows for facile visual assessment of live cell cultures. Protein low-fouling polymer brushes could be developed into commercially viable culture surfaces for hPSC maintenance and also for novel differentiation protocols because radical polymerisation methods allow them to be inexpensively and consistently produced at scale. Further, polymer brushes can be applied to a range of materials including surfaces with complex geometries, which make them relevant to potential future applications including microcarrier suspension cultures, which are discussed below.

### Three dimensional (3D) hPSC culture systems

3D culture systems include hydrogels and suspension cultures and are of increasing interest to the field due to the economies of scale involved. These culture systems produce superior expansion rates and yields to traditional two dimensional (2D) culture systems. While the more immediate applications for hPSCs are likely to be drug screening and disease modelling, which require fewer cells and could be approached with conventional 2D culture systems; clinical applications demand higher cell numbers and more stringently defined culture conditions for reasons of safety. Well-optimised 3D culture systems are therefore expected to be indispensible for generating the quality and quantity of cells required for clinical applications of hPSCs. For example, repair of a typical myocardial infarction has been estimated to require 1–2 billion (x10^9^) cells [108]. Standard two dimensional (2D) culture systems routinely produce cell densities of ~2x10^5^ cells/cm^2^ such that 5000-10000 cm^2^ of culture surface would be needed to generate enough cells to treat a single myocardial infarction [109] (see Table 3 for costings). Conversely, suspension culture of hPSCs has been reported at culture densities of 6.1x10^6^cells/ml and producing a 20-fold expansion in 7 days compared to the 11.3-fold expansion for 2D culture [110]. In order to generate the requisite number of hPSCs to repair a single myocardial infarction using a multi-stage bioreactor system a much more manageable 200 ml culture volume would be needed [111].

**Table 3:** **Culture surface coating requirements and costing for the generation of 1 billion (x10** ^**9**^ **) hPSCs**

Surface	Manufacturer	Coating density source	Cost per cm^2^($USD)^1^	Cost per 1 trillion cells (5000 cm^2^) ($USD)
Matrigel^TM^	Becton Dickinson	Becton Dickinson handbook	$0.080	$400
	Cat No: 354277			
Geltrex^TM^	Invitrogen	Life Technologies^TM^ handbook	$0.062	$310
	Cat No: A1413302			
Recombinant human laminin-511 (whole protein)	BioLamina	[112]^2^	$10.7	$53331
	Cat No: LN511			
Recombinant human vitronectin (truncated protein)	Gibco®	[73]	$0.041^3^	$205
	Cat No: A14701SA			
Recombinant human fibronectin	Abcam	[113]	$0.664	$3320
	Cat No: AB92798			
Corning Synthemax^TM^ II-SC	Corning Inc.	Corning handbook	$0.205	$1026
	Cat No: 3535XX1			
StemAdhere^TM^(E-cadherin fusion protein)	Primorigen Biosciences ® Cat No: S2112	Primorigen handbook	$0.081	$406

Calculations were based on a typical cell density of 2x105 cells/cm2. These calculations do not take into account requirements for media or plasticware or the implementation of cardiomyocyte differentiation protocols. It should also be noted that such protocols are not 100% efficient, so it is likely that additional cells would be required.

^1^Prices were obtained from the websites of Australian suppliers of the manufacturers listed and converted from $AUD to $USD on the ninth of April 2013 ($1AUD =1.04$USD).

^2^Only concentrations of ECMPs in solution were reported so a volume of 50ul/cm^2^ was used for calculations, based on recommendations for Matrigel^TM^ coatings (Becton Dickinson).

^3^At the time of writing recombinant vitronectin was being promoted in combination with the hPSC media E8 and as such was being sold at low cost.

#### Hydrogels

Hydrogels are three-dimensional structures composed of cross-linked, hydrophilic, polymeric scaffolds which expand into an ECM-like gel state when exposed to water. Hydrogels have a wide range of potential uses in tissue engineering [reviewed in [114]. For cell culture thin hydrogels can be used as an effectively 2D culture substrate where the cells are present on the surface only or thicker hydrogels can be used to encapsulate cells, allowing them to proliferate and migrate within the gel [114]. Like their constituent polymers, hydrogels can be inherently bioactive or relatively inert. Hydrogels can also be composed of multiple polymer chains which modify the physical properties of the gel or incorporate cell-binding motifs [114].

Hyaluronic acid- [67, 115] and polyacrylamide-based hydrogels [15] as well as amino-propylmethacrylamide hydrogels [14] have been demonstrated to support long-term culture of hPSCs. These hydrogels interact with hPSCs through non-specific protein adsorption [14], pre-coated ECMPs [15] or combinations of the two [67]. Hydrogels incorporating sulfonyl-containing polymer chains like PSS(S) have also been shown to support long term culture of hPSCs, presumably by mimicking HSPGs [102]. Interestingly hydrogels incorporating PMEDSAH failed to support hPSC adhesion as did several other sulfonyl-containing hydrogels [102]. With respect to hydrogels presenting specific cell-binding ligands, semi-interpenetrating polymer network hydrogels presenting an RGD-motif have been shown to enable hPSC-binding [116]. However these gels were also protein-fouling, used with MEF-CM and were not tested in longer-term culture [116]. A (meth)acrylate-based hydrogel modified to present vitronectin-derived RGD-containing peptides is currently commercially available as the hPSC culture surface Corning Synthemax^TM^ although the protein fouling properties of this hydrogel have not been published. A study using a polyacrylamide hydrogel modified with a vitronectin-derived HSPG-binding peptide recently demonstrated the first example of long-term self-renewal of hPSCs capable of *in vitro* differentiation into the three germ layers, cultured on protein low-fouling, peptide-presenting hydrogels with modifiable physical properties in defined medium [92]. This provides an interesting tool for controlling self-renewal of hPSCs in a chemically-defined hydrogel culture system.

#### hPSC suspension culture systems

Suspension culture systems involve either cell-adherent microcarriers [117] or free-floating clumps of hPSCs [111] that are held in suspension by either shaking or stirred suspension bioreactors. The details of this area are beyond the scope of this review and have been well-reviewed elsewhere [118, 119] but the potential applications of these culture systems cannot be ignored. Briefly, current challenges for hPSC suspension culture systems include reducing shear forces, maintaining even cell distribution across microcarriers and passaging expanding cell aggregates [118]. Due to the potential gains and economies of scale involved, all work on hPSC culture surfaces and CAM/ECMP interactions should be undertaken with adaptation to fully-defined microcarrier-based suspension culture systems in mind. Protein coatings and polymer scaffolds can also be readily adapted to microcarrier -based suspension culture systems [120].

### Comparisons of hPSCs cultured on different surfaces and future directions

Studies comparing defined surfaces have so far been focused on identifying culture conditions that were equally effective as MEF- or Matrigel^TM^-based systems at reaching the fundamental goals of hPSC culture such as promoting cell adhesion, proliferation and expression of key pluripotency markers [15, 16, 74]. These studies used different combinations of surfaces and media without performing comprehensive cross-comparisons [16], were more focused on optimising culture media [17] and/or did not characterise and compare the cells at a high level of detail. Many of these studies also used whole ECMPs with multiple binding sites so they were unable to investigate the role of CAMs in maintaining pluripotency, and little characterisation of surface properties was performed. Some recent studies have included more detailed characterisation of hPSCs as they are cultured in various complex, undefined culture systems and have identified genetic and epigenetic changes that take place during adaptation to changes in culture conditions [121, 122]. While the phenotypic changes were fairly minor and their causes and/or effects unclear, greater variation could be expected between the products of chemically-defined culture systems. It is therefore important that both cell and surface are characterised thoroughly during development of culture systems. Now that a range of defined culture surfaces have been reported to support maintenance of hPSCs to a level equivalent to Matrigel^TM^ by the minimum criteria and that the ligands involved have been identified, it should be possible to compare the resulting cells in more detail and to optimise surfaces for hPSC culture systems by taking into consideration the importance of CAM-mediated interactions, of physicochemical surface properties and of cost. Other future directions for defined culture surfaces associated with hPSCs include applications to directed differentiation protocols and to the reprogramming of somatic cell types as changes to CAM expression have been identified during differentiation [28] and reprogramming [123]. Identification of these changes and their role(s) in differentiation should be instructive for current approaches to developing inductive biomaterials for differentiation protocols {comprehensively reviewed in [124]}.

## Conclusions

Pluripotency is a complex state maintained *in vitro* by molecular signals received from the cellular microenvironment, including the cell culture surface. Commonly implemented hPSC culture surfaces are composed of complex animal products presenting undefined ligands that interact with many CAMs on the cell surface and transmit pluripotency-supporting molecular signals to the nucleus [3–5]. hPSC culture surfaces have evolved from xenogenic feeder cell layers with complex cell-surface interactions and no potential for use at scale to purified human recombinant ECMPs including laminin, fibronectin, vitronectin and HSPGs. Such ECMP-coated surfaces are xeno-free and interact with hPSCs through smaller sets of CAM/ligand interactions that are ECMP-specific, however production of recombinant proteins is expensive. Furthermore, synthetic surfaces are being developed based on the functional subunits of ECMPs and these surfaces have been reported to maintain pluripotency through more specific interactions (see Table 4 for examples). Defined surfaces thought to specifically interact with hPSCs through different CAMs appear to be equally supportive of culture, although direct and detailed comparisons between hPSCs maintained on surfaces that interact with different CAMs have not been performed. Identifying the role(s) of CAMs in maintaining pluripotency will be an important step towards developing defined, affordable, xeno-free culture conditions for hPSCs suitable for clinically relevant large scale hPSC culture.

**Table 4:** **Ligand-CAM interactions reported to support long-term hPSC culture**

Substrates	Ligands or ECMPs	CAM(s)	References
Tissue culture polystyrene (TCP)	Vitronectin	αVβ3/5 integrins, GAGs	[7]
	Laminin-511	α6β1 integrin	[11]
	Laminin E8 fragments	-	[13]
	Fibronectin	α5β1 integrin	[48]
	Collagen + fibronectin + laminin + vitronectin	-	[125]
	Poly(L-lysine)	-	[126]
	E-cadherin-IgG1Fc (StemAdhere^TM^)	E-cadherin	[10]
Amine-modified TCP	Cyclic-CRGDC	-	[86]
UV-treated TCP	Adsorbed serum proteins, vitronectin	-	[12]
Acrylate monomer-coated TCP	KGGNGEPRGDTYRAY	αVβ5	[9]
	(Corning Synthemax^TM^)	integrins - αVβ3/5	[9]
	KGGPQVTRGDVFTMP		
	Vitronectin	integrins, GAGs	[42]
Self-assembled monolayers	GKKQRFRHRNRKG	HSPGs	[79]
	LTTAPKLPKVTR	GAGs	[127]
Amino-propylmethacrylamide	BSA + non-specific proteins (adsorbed from media)	-	[14]
Hydrogels			
Polyacrylamide hydrogel	GKKQRFRHRNRKG	HSPGs	[92]
PMEDSAH	Unknown. Adsorbed growth factors?	-	[99]

Surfaces are arranged according to their substrate. The ligands or extracellular matrix proteins (ECMPs) that are presented from those substrates and the CAMs with which they have been shown to interact (if any) are also listed. Whole ECMPs, ECMP fragments, fusion proteins, and peptides presented by amine-modified or acrylate monomer coated TCP, protein-fouling hydrogels and polymers have all demonstrated the capacity to support hPSC culture by interacting with various integrins, E-cadherins and/or heparan sulphate proteoglycans. The surfaces listed have all been reported to support hPSC culture subject to at least the minimum cell characterisation requirements outlined in Table 1. Key references have been provided for each surface.

## Abbreviations

2D: Two dimensional
3D: Three dimensional
AT: Alkanethiol
CM: Conditioned media
ECMPs: Extracellular matrix proteins
CAM: cell adhesion molecule
ESCs: Embryonic stem cells
FISH: Fluorescence *in situ* hybridization
FGF-2: Basic fibroblast growth factor
GAGs: Glycosaminoglycans
hPSCs: Human pluripotent stem cells
HS: Heparan sulphate
HSPGs: Heparan sulphate proteoglycans
iPSCs: Induced pluripotent stem cells
MEF: Mouse embryonic fibroblast
mPSCs: Murine pluripotent stem cells
NC-CM: Neonatal chondrocyte-conditioned medium
NMR: Nuclear magnetic resonance
PAMPS: Poly(acrylamido-methyl-propane sulfonate)
PLGA: Poly (lactic-co-glycolic acid)
PMVE-alt-MA: Poly(methyl vinyl ether-alt-maleic anhydride)
PSS(S): Poly(sodium 4-styrenesulfonate)
SAMs: Self assembled monolayers
SNP: Single-nucleotide polymorphism
TCP: Tissue culture polystyrene.
